# Effectiveness of Nanohydroxyapatite on Demineralization of Enamel and Cementum Surrounding Margin of Yttria-Stabilized Zirconia Polycrystalline Ceramic Restoration

**DOI:** 10.1155/2021/5540738

**Published:** 2021-05-19

**Authors:** Niwut Juntavee, Apa Juntavee, Preeyarat Plongniras

**Affiliations:** ^1^Department of Prosthodontics, Faculty of Dentistry, Khon Kaen University, Khon Kaen 40002, Thailand; ^2^Division of Pediatric Dentistry, Department of Pediatric Dentistry, Faculty of Dentistry, Khon Kaen University, Khon Kaen 40002, Thailand; ^3^Division of Biomaterials and Prosthodontics Research, Faculty of Dentistry, Khon Kaen University, Khon Kaen 40002, Thailand

## Abstract

**Introduction:**

Prosthetic dentistry has shifted toward prevention of caries occurrence surrounding restorative margin through the anti-demineralization process. This study examines the ability of nanohydroxyapatite (NHA) gel and Clinpro (CP) on enhancing resistance to demineralization of enamel and cementum at margin of restoration.

**Materials and Methods:**

Thirty extracted mandibular third molars were segregated at 1 mm above and below cementoenamel junction (CEJ) to separate CEJ portions and substituted with zirconia disks by bonding to crown and root portions with resin adhesive. The enamel and cementum area of 4 × 4 mm^2^ neighboring zirconia was applied with either NHA or CP, while one group was left no treatment (NT) before demineralized with carbopal. Vickers hardness (VHN) of enamel and cementum was evaluated before material application (*B*_M_), after material application (*A*_M_), and after demineralization (*A*_D_). Analysis of variance (ANOVA) and post hoc multiple comparisons were used to justify for the significant difference (*α* = 0.05). Scanning electron microscopy (SEM) and X-ray diffraction (XRD) were determined for surface evaluations.

**Results:**

The mean ± SD of VHN for *B*_M_, *A*_M_, and *A*_D_ for enamel and cementum was 393.24 ± 26.27, 392.89 ± 17.22, 155.00 ± 5.68 and 69.89 ± 4.59, 66.28 ± 3.61, 18.13 ± 0.54 for NT groups, respectively, 390.10 ± 17.69, 406.77 ± 12.86, 181.55 ± 7.99 and 56.01 ± 9.26, 62.71 ± 6.15, 19.09 ± 1.16 for NHA groups, respectively, and 387.90 ± 18.07, 405.91 ± 9.83, 188.95 ± 7.43 and 54.68 ± 7.30, 61.81 ± 4.30, 19.22 ± 1.25 for CP groups, respectively. ANOVA indicated a significant increase in anti-demineralization of enamel and cementum upon application of NHA or CP (*p* < 0.05). Multiple comparisons indicated the capability in inducing surface strengthening to resist demineralization for enamel and cementum of NHA which was comparable to CP (*p* > 0.05) as evidenced by SEM and XRD data indicating NHA and CP deposition and crystallinity accumulation.

**Conclusion:**

NHA and CP were capable of enhancing anti-demineralization for enamel and cementum. The capability in resisting the demineralization process of NHA was comparable with CP. NHA was highly recommended for anti-demineralization for enamel and cementum surrounding restorative margin.

## 1. Introduction

Ceramic has been considerably increasing as a preferable restorative material for contemporary aesthetic dentistry. The advancement in digital dentistry based on the technological development of computer-assisted design-computer-assisted manufacturing (CAD-CAM) has been accepted by dental practitioners in search of novel restorative materials, which are capable of providing superior aesthetics quality and durable restorations together with an excellent prognosis. Several dental ceramics have been introduced to satisfy the demands from both the dentists and patients for construction and the restoration with extremely aesthetic and natural tooth appearance. Many innovative ceramic-comprised materials have been developed for fabrication of the restorations with the technology of CAD-CAM comprising hybrid ceramic, feldspar ceramic, leucite-reinforced ceramic, lithium disilicate glass ceramic, zirconium-reinforced lithium silicate ceramic, yttria partially stabilized tetragonal zirconia polycrystal (Y-TZP) ceramic, and monolithic zirconia [1]. These ceramics were innovated with strengthening properties to exert physiologic load from the mastication system. Nevertheless, the preciseness of all-ceramic restoration is considerably less compared to ceramometal restoration [2]. The imprecise fit of restoration engenders bacteria accumulation in the dental plaque, which instigates gingival inflammation and demineralization of enamel causing restoration failure [2]. Imprecise marginal fit of ceramic restoration to prepared tooth abutment often causes demineralization of tooth structure around the restorative margin, which is usually situated on the surface of enamel adjacent to the cementoenamel junction (CEJ). Nonetheless, the margin of restoration is sporadically settled on the surface of cementum, beyond the CEJ in the extensive restoration of the compromised periodontium in advanced fixed prosthodontic treatment. Successful restorations depend on the preciseness of the margin of the restoration to be conformed with the prepared tooth, which is usually located either on enamel or cementum.

Tooth decay is a common infectious disease involving carbohydrate-modifying bacteria in humans, leading to the destruction of tooth structure. Dental caries is an extremely established multifactorial infection, affecting a large percentage of world population and generates a substantial financial problem worldwide [3]. The fundamental execution of tooth decay relates to demineralization via the aggression by acids produced from bacterial biofilms. The formation of dental biofilm is essential for bacterial growth to expose to fermentable carbohydrates, producing acids that are responsible for demineralization [4]. A number of dental restorations were placed annually with increasingly used ceramic restorations. The restorations are attached to the natural tooth abutments with resin cement, which seem to induce bacterial biofilm agglomeration over other restorative materials [5]. The bacterial biofilm adhering to the restorative margins can expedite the development of demineralization surrounding the restorative margin and shorten the lifetime of the restoration. Recurrent caries nearby the margin of restoration have been contemplated as a principal reason for failure of restorative treatment, therefore, reducing the durability of restorations [6]. It was described that approximately 18–22% of the abutments for fixed dental restorations were affected by dental decay, causing the endodontic treatment and further replacing with new restorations [7]. It is generally accepted that an appraisement of recurrent caries beneath the margin of restoration is considerably problematic [8]. Initial demineralization surrounding the margin of restoration either on cementum or on enamel is scarcely noticeable by a radiographic method. Deferred determination of recurrent caries beneath the margin of restoration engenders irreversible pulpal infection [8]. Decay surrounding the dental restorative margin is reasonably related to the microleakage of dental restoration and dissolution of the cementation material [9]. It was described that the occurrence of root caries is extremely high, and it ranges approximately 7.3–69.7% [10–12].

The inhibition of demineralization occurrence surrounding the margin of the restoration was denoted by the theory of anti-demineralization and remineralization of a natural tooth. Anti-demineralizing materials have been introduced into dentistry in different forms, for instance, restorative materials, pit and fissure sealants, mouth rinses, toothpaste, and chewing gums [13, 14]. Fluoride has been described as an utmost efficient material for caries prevention. Nonetheless, excessive fluoride uptake, causing fluorosis as well as systemic toxicity, becomes a major concern [13]. Recently, alternative materials to fluoride were recommended, comprising nanohydroxyapatite (NHA) and casein phosphopeptide amorphous calcium phosphate (CPP-ACP) due to its anti-demineralization capabilities [5, 14, 15]. The hydroxyapatite has gained much attraction for prosthodontic applications because of its composition (comprising calcium and phosphate) and the crystalline structure comparable to human's bone and tooth. Recently, the nanoparticle of hydroxyapatite has been receiving much attention in medicine and dentistry. The nanosized hydroxyapatite was applied for the treatment of osteoporosis, periodontitis, and severe residual ridge resorption [16–18]. The NHA was described to be capable of preventing and the initial therapeutic approach for dental decay, especially in controlling biofilm of dental plaque and remineralization capability for initial carious lesion [19]. Nanomaterials are usually determined by the size of the particle that is decreasing in size from micrometers to nanometers, which result in drastic changing in both physical and mechanical properties, for instance, surface hardness, surface affinity, and facilitating both chemical and biological activities [20]. Nanomaterials have been introduced as a preventive aspect of prosthetic dentistry in the concept of anti-demineralizing products [19]. Numerous anti-demineralization products are available for dental use to enhance tooth structure to resist acid attack from the demineralization process [21, 22]. The NHA is an extreme bioactive and biocompatible material for the purpose of strengthening tooth structure to be hardly demineralized as well as initiating the remineralized process [19, 23]. NHA also possesses antibacterial property as described by another study [24]. Several studies reported that 10% NHA is capable of efficiently resisting demineralization on dental enamel [14, 25, 26]. Some studies described an equivalent or better inhibition of demineralization by NHA toothpaste compared to other toothpastes comprising of fluoride and aminofluoride [27, 28]. Several studies described that NHA of particle size 60–80 nm in length and 10–20 nm in diameter was better for penetration into the interprismatic space through the ion transportation process. This is probably connected to the interprismatic protein, developing anti-demineralization on the outermost layer of caries lesions and conceivably converse the advancement of an initial carious process [29–34]. Nevertheless, to date, there is no report on the use of any NHA in gel form for the preventive prosthodontic treatment. The purpose of this study is to assess the utility of a recently developed product in the form of NHA gel, containing 10% nanohydroxymethyl cellulose (SCMC) for anti-demineralization on the surface of cementum and enamel nearby the margin area of Y-TZP restorative dental ceramic.

## 2. Materials and Methods

The study was endorsed by Khon Kaen University Ethical Committee in Human Research (approved no. : HE 592239) and followed the CRIS guidelines for in vitro studies. All patients whose extracted teeth were used for this study provided a written informed consent.

### 2.1. Specimen Preparation

**Figure 1:**
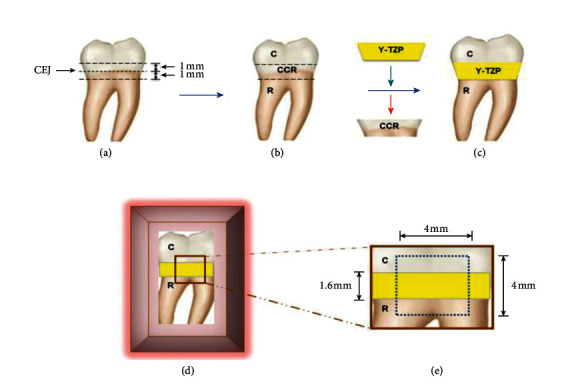
Human third molar (a) was sectioned at 1 mm below and above cementoenamel junction (CEJ) (b). The crown-CEJ-root (CCR) portion was removed and replaced with the yttria-stabilized tetragonal zirconia polycrystalline (Y-TZP) disk by bonding to the crown (C) and root (R) portion using resin cement (c) and then inserted in acrylic block (d) to create a flat surface area of 4 × 4 mm^2^ (e).

Thirty extracted mandibular third molars were included in the experiment. The teeth were cut with an exactness instrument (Isomet 4000®, Buehler, IL, USA) at the distance of 1 mm below and above the CEJ to divide into 3 portions including crown (C), crown-CEJ-root (CCR), and root (R) (Figure 1(a)). The CCR piece was separated and used as a model for the production of a zirconia disk (thickness = 2 mm) (Figure 1(b)). A partially sintered Y-TZP blank (inCoris TZI, Dentsply, York, PA, USA) was milled into a form of a disk at a comparable shape with the CCR, except for 20% larger in dimension to compensate for sintering shrinkage of zirconia. Each zirconia disk was fired in the high-temperature furnace (inFire HTC speed, Dentsply), just as the manufacture's recommendation at 1510°C for 2 hours, to originate an exact 1.6 mm thickness with absolutely analogous CCR contour. The zirconia disk was jointed in between the C and R parts using autopolymerized adhesive resin (Superbond C and B, Sun Medical, Shiga, Japan) at an exact cement thickness of 25 microns, utilizing a digital vernier caliper (Mitutoyo, Chicago, IL, USA) (Figure 1(c)). The specimen was inserted in the resin block (Unifast Trad, GC, Tokyo, Japan), and the surface was left exposed for further experimentation (Figure 1(d)). A smooth area of 4 × 4 mm^2^ was produced on the exposed surface area of a specimen, using an automatic polisher (Ecomet 3, Buehler, Lack Buff, IL, USA) (Figure 1(e)). The specimen was rinsed with spray water and immersed in the 37°C deionized water for 24 hours.

### 2.2. Application of the Anti-Demineralization Materials

The specimens were randomly separated into three groups (*n* = 10) and applied with either nanohydroxyapatite (NHA) gel or sodium fluoride tooth cream (0.21% w/w, Clinpro; CP, 3M-ESPE, St. Paul, MN, USA), while one group was left in deionized water (no treatment) and served as a control group (NT). Each anti-demineralizing agent was smeared to the surface of cementum and enamel neighboring zirconia margin, covering the area 4 × 4 mm^2^ for 4 minutes before soaking in newly prepared deionized water. The anti-demineralized materials were smeared two times a day, at an interval of 12 hours, for 30 days.

### 2.3. Induction of Artificial Caries Lesion

The demineralizing agent used for inducing artificial caries lesion was prepared in a synthetic polymer gel, comprising of 20 grams/liter of Carbopol 907 (BF Goodrich, Cleveland, OH, USA), and combined with 0.1% lactic acid and 0.2% polyacrylic acid, and then regulated to the pH of 4.4 by sodium hydroxide [35]. All specimens were submerged in a demineralizing gel, kept in the humid atmosphere for 16 hours to produce caries lesion on both cementum and enamel, and then cleaned with deionized water to eradicate the residual gel on the specimen surface.

### 2.4. Evaluation of Surface Hardness

**Figure 2:**
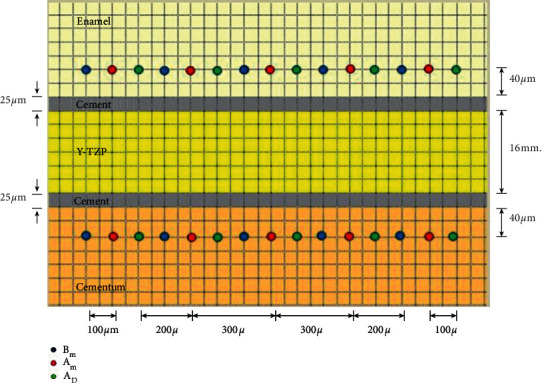
Vickers microhardness of enamel and cementum was determined before material application (*B*_M_), after material application (*A*_M_), and inducing demineralization (*A*_D_) (5 locations each; with 100 microns apart) at 40 microns from enamel-resin cement junction and cementum-resin cement junction.

Vickers microhardness of cementum and enamel was determined by depressing with the Vickers diamond indenter at 100 grams load for enamel and 10 grams load for cementum with 15 seconds indenting time using a microhardness tester (Digital Hardness Tester FM-800, Future-Tech, Tokyo, Japan). The hardness of cementum and enamel was evaluated at the span of 40 microns below the cementum-cement junction and above the enamel-cement junction. Hardness was evaluated before application of anti-demineralizing material (*B*_M_), to serve as the reference data baseline, after application of anti-demineralizing material (*A*_M_), and after inducing demineralized lesion (*A*_D_). Each indentation was randomly determined at 100 microns apart from each other (Figure 2).

### 2.5. Statistical Analysis

The data were statistically analyzed using Statistic Package for Social Science (SPSS, Chicago, IL, USA). An analysis of variance (ANOVA) was performed to conclude for a significant difference of Vickers hardness of different anti-demineralization materials at each stage of exploration involving *B*_M_, *A*_M_, and *A*_D_ for both cementum and enamel. Tukey HSD multiple comparisons were used to evaluate for the difference between each factor at 95% confidential interval.

### 2.6. Scanning Electron Microscope Photomicrograph

The samples from each group were investigated for the alterations of cementum and enamel surface neighboring the zirconia at each phase of investigation including *B*_M_, *A*_M_, and *A*_D_ phases. The samples were gold coated in sputtering equipment (Emitech K-500X, Quorum Technology, Ashford, UK) and examined in the scanning electron microscope (SEM-S3000 N, Hitachi, Tokyo, Japan).

### 2.7. Evaluation of Crystalline Structures

The samples from each group were randomly designated to evaluate for crystal structures both in the cementum and enamel by an X-ray diffractometer (XRD, PANalytical-BV, Almelo, Netherlands). The samples were milled into a fine powder and then serially scanned with copper k-alpha (Cu K-*α*) radiation by 30 mA, 40 kV at 2*θ* degree at 20–60°. The crystal structure was justified by comparing with the known standard crystalline structure provided by the joint committee on powder diffraction standard (JCPDS) and was examined for the peak intensity using Xpert plus software (PANalytical-BV) at 0.02° step size for every 2 seconds.

## 3. Results

**Figure 3:**
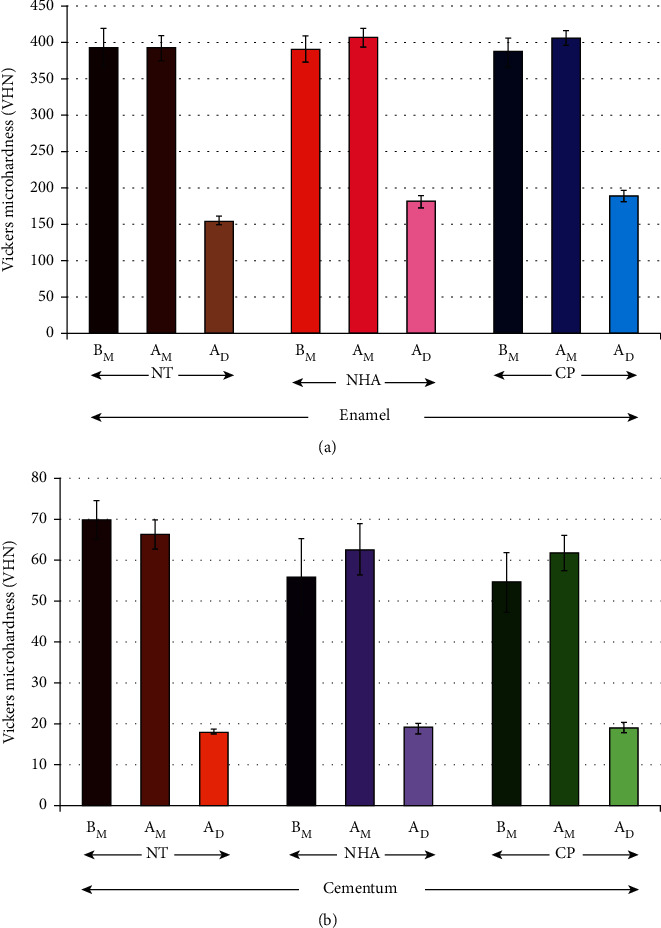
The mean and standard deviation (SD) of Vickers microhardness (VHN) of enamel (a) and cementum (b) before material application (*B*_M_), after material application (*A*_M_), and after inducing demineralization (*A*_D_) with nanohydroxyapatite (NHA) and Clinpro (CP), in comparison with no treatment (NT).

The results of the study on the anti-demineralization potential of NHA for both the cementum and enamel neighboring the marginal area of zirconia restoration found on surface hardness are presented in Table 1 and Figure 3. The means ± SD of microhardness for NT groups at *B*_M_, *A*_M_, and *A*_D_ were 393.24 ± 26.27, 392.89 ± 17.22, and 155.00 ± 5.68 for enamel and 69.89 ± 4.59, 66.28 ± 3.61, and 18.13 ± 0.55 for cementum, respectively. The means ± SD of microhardness for NHA groups at *B*_M_, *A*_M_, and *A*_D_ were 390.10 ± 17.69, 406.77 ± 12.86, and 181.55 ± 7.99 for enamel and 56.01 ± 9.26, 62.71 ± 6.15, and 19.09 ± 1.16 for cementum, respectively. The means ± SD of microhardness for CP groups at *B*_M_, *A*_M_, and *A*_D_ were 387.90 ± 18.07, 405.91 ± 9.83, and 188.95 ± 7.43 for enamel and 54.68 ± 7.30, 61.81 ± 4.30, and 19.22 ± 1.25 for cementum, respectively.

ANOVA denoted that there were significant differences in microhardness for both the cementum and enamel area neighboring the zirconia restoration due to the different anti-demineralizing materials, phases of material application, as well as the interaction between material and stage of application (*p* < 0.05), as presented in Table 2. Post hoc multiple comparisons illustrated a significant ability to increase hardness to the surface of either cementum or enamel neighboring the zirconia restorations for both CP and NHA in comparisons to the NT group (*p* < 0.05), as presented in Table 3. Furthermore, a significantly high capability of both enamel and cementum treated with either NHA or CP in resisting demineralization in comparisons to no treatment (*p* < 0.05) is shown in Table 3. Both NHA and CP indicated no significant differences in the capability to strengthen surfaces or to resist demineralization to the surfaces of either cementum or enamel (*p* > 0.05), as given in Table 3.

**Figure 4:**
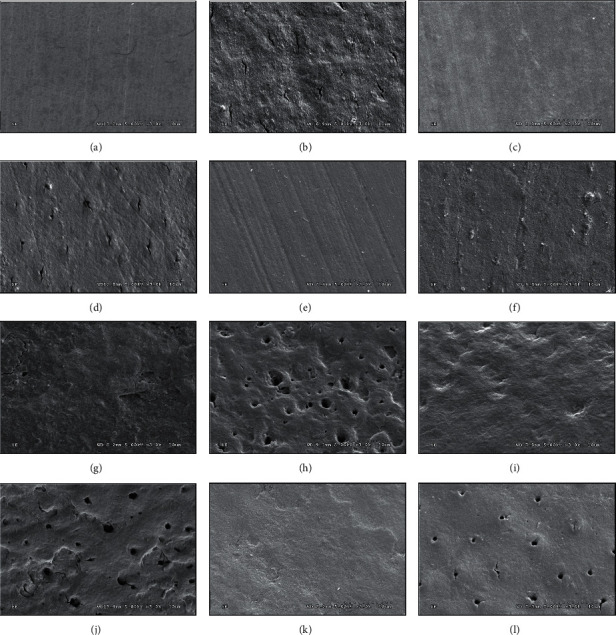
Scanning electron microscopy (SEM) of enamel (a, c, e, g, i, k) and cementum (b, d, f, h, j, l) before material application (a, b), after surface applied with NHA (c, d), and Clinpro (e, f) and after demineralization of nontreated surface (g, h), NHA treated surface (i, j), and Clinpro treated surface (k, l).

The SEM micrograph indicated generalized smooth architecture for both enamel (Figure 4(a)) and cementum surfaces (Figure 4(b)). The surface architectures of either enamel or cementum revealed a smoother surface architecture applied with either NHA (Figures 4(c) and 4(d)) or CP (Figures 4(e) and 4(f)) compared to the untreated surface of either enamel (Figure 4(a)) or cementum (Figure 4(b)). Widespread surface irregularities of demineralized enamel (Figures 4(g), 4(i), and 4(k)) and widespread irregularities of demineralized cementum with opening tubules (Figures 4(h), 4(j), and 4(l)) were denoted after the demineralized process. The demineralized surface of untreated enamel (Figure 4(g)) and untreated cementum (Figure 4(h)) exhibited more irregularities on the demineralized surfaces with larger size of the opening tubules in the cementum compared to the demineralizing surfaces of either enamel or cementum previously treated with either NHA (Figures 4(i) and 4(j)) or CP (Figures 4(k) and 4(l)). The SEM photomicrograph illustrated the accumulation of NHA particles in the cementum tubules, together with a minimal surface irregularity (Figure 4(j)). Contrarily, some particles of CP appeared to precipitate in the tubules of cementum with a minor area of irregularity on the surface (Figure 4(l)).

**Figure 5:**
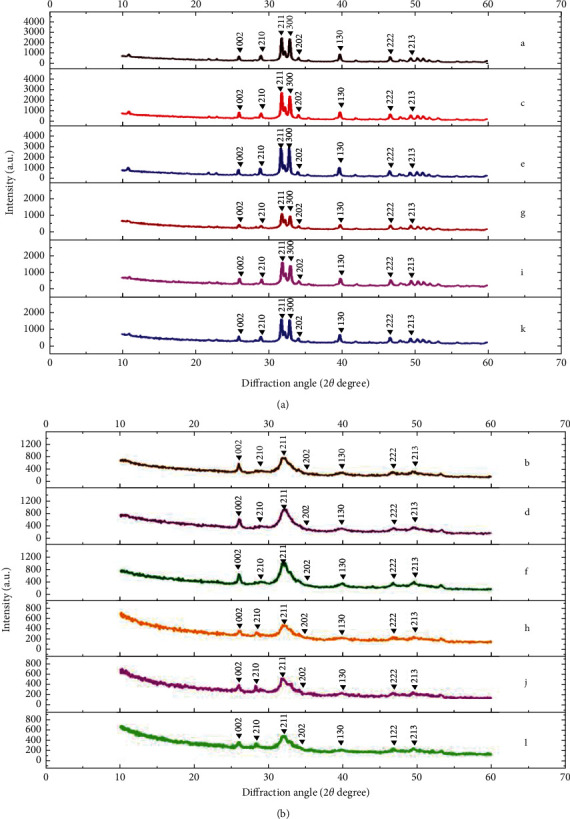
The X-ray diffraction (XRD) pattern of enamel (a) and cementum (b) before material application (a, b), after surface applied with NHA (c, d), and Clinpro (e, f) and after demineralization to a nontreated surface (g, h), NHA treated surface (i, j), and Clinpro treated surface (k, l).

The XRD revealed that the crystalline structures remarked at the 2*θ* degree of 26°, 29°, 31°, 32°, 33°, 34°, 39°, 46°, 49°, 51°, and 52°, matched with the (002), (210), (211), (112), (300), (202), (130), (222), (213), (321), and (140) plane for both enamel and cementum for all groups. Nevertheless, the main peak with the strongest intensity was detected at 2*θ* degree of 31° and 33°, which matched with the (211) and (300) plane for both the enamel and cementum (Figures 5(a) and 5(b)). The intensity of peak was higher in enamel than in cementum (Figures 5(a) and 5(b)). The intensity of peak increased upon application of anti-demineralizing materials with either NHA for approximately 22.17% and 19.30% in enamel and cementum (Figures 5(c) and 5(d)) or CP for 15.32% and 16.80% in enamel and cementum (Figures 5(e) and 5(f)) compared to nontreated enamel and cementum. The peak intensities decreased upon the demineralization process by approximately 53.36% and 46.99% for untreated enamel and cementum (Figures 5(g) and 5(h)), 41.62% and 44.30% for NHA treated enamel and cementum (Figures 5(i) and 5(j)), and 44.22% and 47.93% for CP-treated enamel and cementum, respectively (Figures 5(k) and 5(l)). The peak intensities of demineralized NHA- and CP-treated enamel were approximately 59.92% and 37.92% higher than the demineralized untreated surface, respectively, as well as that for approximately 14.06% and 4.46% in demineralized treated cementum, respectively.

## 4. Discussion

Absolute consolidation of junction between the restoration and the prepared tooth surface is a supreme goal of fixed prosthodontic treatment that is hardly achieved. Certainly, an imperfect adaptation of restoration at the margin and microgaps at the interfaces of the tooth restoration always exists which promotes bacteria to accumulate surrounding the margin of the restoration, leading to dental caries at the margin of the restoration. Therefore, the concept of caries inhibition at a restorative junction with tooth structure is considered a new paradigm for contemporary prosthetic dentistry. The study determined the capability of NHA and CP on strengthening cementum and enamel to resist the demineralization process at the cavosurface area surrounding the zirconia. This study demonstrated that both NHA and CP were significantly able to strengthen both enamel and cementum surfaces to resist the demineralization process, compared to the nontreated surface. The study indicated that the potential of NHA in enhancing the anti-demineralization for both cementum and enamel is comparable to that of CP, as evidenced from SEM which indicated NHA and CP deposition in the surface areas of enamel and cementum. Also, XRD displayed accumulative peak intensities to cementum and enamel upon NHA or CP application. This designated a probable mechanism of the ion transport for the anti-demineralization procedure [29]. The matrix protein comprised approximately 1% of the organic part in mature enamel, which probably remained and functioned as the key scaffolding for ionic transmission [30] and accumulation in the nanogaps of the interprismatic area, as explained in other reports [30, 31, 34]. The enamel-proteins positioned in the interprismatic area were conceivably capable of catching the mineral contents and permitted the diffusion of minerals along the sides of the crystalline structures [29, 31]. This is additionally associated with the isomorphic and isoionic exchanging processes in enamel crystal structures and may have facilitated the transmission process of phosphate and calcium through interprismatic areas and turned to hydroxyapatite structures [29]. These proteins possibly worked as a scaffold for ion exchange of CP and NHA [22, 31]. This results in the abilities of CP and NHA gel to enhance the anti-demineralization phenomenon [13]. However, NHA seems to indicate a slightly more capable in the anti-demineralization process than CP. It is probably associated with the nanoparticle of NHA, which is susceptible to the microstructure of the tooth. NHA is capable of improving penetration of its crystal through the interprismatic space of enamel which results in the establishment of the hydroxyapatite crystal structures [29, 34]. The sodium fluoride and tricalcium phosphate-containing approximately 0.21% by weight in CP may face more complexity to form crystallization to stimulate surface anti-demineralization. Whereas, the fluoride, calcium, and phosphate compositions in CP could only be forming fluoroapatite crystal in enhancing surface resistance to the anti-demineralization process, as evidenced from SEM which indicated a higher amount of NHA particles than CP particles remaining on the surface of demineralized enamel and in the tubules of cementum, as supported by another study [22].

The surface architecture of enamel was remarkably irregular and somewhat spongy [10]. Such surfaces enabled the NHA to diffuse along with interprismatic areas through the precipitating mechanism. Furthermore, it also induced a large quantity of Ca^2+^ and (PO_4_)^3−^ from the saturated NHA around the external surface of the enamel to fill in the unoccupied positions in the crystal structures [23, 25]. The demineralized cementum was observed obviously with the opening tubules on the surface. However, higher amounts of NHA depositions in the tubules were found as a result of enhancing the surface anti-demineralization effect [10, 11]. The potential for anti-demineralization of cementum as using either NHA or CP is probably associated with the interchanging of minerals between cementum surface and surrounding environment as established in the case of enamel. Nevertheless, the anti-demineralization process in cementum is less strong than in enamel as evidenced from the XRD, which illustrated a shallow intensity of the peak that is probably associated with the lack of protein scaffolding in cementum to promote surface strengthening to resist the demineralization process [23, 33]. However, the robust anti-demineralization capability in cementum for NHA in comparison to CP is probably correlated with the nanoparticle size that is capable of establishing an interdigitation process in the cementum structure [32, 34]. This experiment used deionized water to substitute saliva to eradicate the confounding factor that could be established from some contents, such as amino acid components in salivary protein that probably affect the natural enamel-matrix proteins of tooth specimens [14]. Likewise, mineral contents in artificial saliva could influence perplexing factors during experimentation, as the deproteinization of enamel significantly provokes degradation of enamel characteristics and endurance [30, 32]. Nevertheless, the experiment showed that NHA empowered anti-demineralization capability for cementum and enamel neighboring ceramic restoration. This novel NHA gel signified propitious anti-demineralization potential for enamel and cementum. Eventhough dentists are capable to deliver the best restorative treatment for their patients, the impreciseness of the restorations to the prepared teeth can still happen, which is considered a challenging situation for this novel product like NHA gel to be capable of preventing decay around the margin of the restoration through the anti-demineralization process. Based on these results, this study advocated favorable preventive prospects in advanced restorative dentistry and is engraved as a new method in decay prevention in contemporary fixed prosthodontic reconstruction.

## 5. Conclusion

The philosophy of using NHA to tackle demineralization through the anti-demineralization process is a relatively new paradigm in the preventive approach of prosthetic dentistry. In consideration of recurrent caries surrounding the marginal area of the restorations as the principal factor for restorative failure, the NHA and CP proved to be effectively capable to provide surface resistance to demineralization for the cementum and enamel neighboring the margin of the restoration, which effectively prevented demineralization. NHA was capable of establishing anti-demineralization, comparable with CP for the anti-demineralization process for both cementum and enamel. The NHA gel seems promising in the preventive prospect of advanced restorative reconstruction to reduce the risk of demineralization of cementum and enamel approximately at the margin of restoration as well as encouraging long-term success.

## 6. Clinical Significance

NHA gel was highly potential in anti-demineralization for enamel and cementum around the restorative margin and was suggested as a preventive approach in restorative reconstruction.

## Figures and Tables

**Table 1:** Mean, standard deviation (SD), and 95% confidence interval of Vickers microhardness (VHN) of enamel (E) and cementum (C) before material application (*B*_M_), after material application (*A*_M_), and after inducing demineralization (*A*_D_) with nanohydroxyapatite (NHA), Clinpro (CP), and no treatment (NT).

1.1 Vickers microhardness of enamel					
Group	*n*	Mean	SD	95% confidence interval	
				Lower limit	Upper limit
E-NT-B_M_	10	393.24	26.27	374.44	412.04
E-NT-A_M_	10	392.89	17.22	380.58	405.21
E-NT-A_D_	10	155.00	5.68	150.94	159.06
E-NHA-B_M_	10	390.10	17.69	377.44	402.76
E-NHA-A_M_	10	406.77	12.86	397.57	415.98
E-NHA-A_D_	10	181.55	7.99	175.83	187.27
E-CP-B_M_	10	387.90	18.07	374.97	400.83
E-CP-A_M_	10	405.91	9.83	398.88	412.94
E-CP-A_D_	10	188.95	7.43	183.63	194.26

1.2 Vickers microhardness of cementum					
Group	*n*	Mean	SD	95% confidence interval	
				Lower limit	Upper limit

C-NT-B_M_	10	69.89	4.59	66.61	73.18
C-NT-A_M_	10	66.28	3.61	63.70	68.87
C-NT-A_D_	10	18.13	0.55	17.74	18.52
C-NHA-B_M_	10	56.01	9.26	49.39	62.63
C-NHA-A_M_	10	62.71	6.15	58.32	67.11
C-NHA-A_D_	10	19.09	1.16	18.26	19.93
C-CP-B_M_	10	54.68	7.30	49.46	59.91
C-CP-A_M_	10	61.81	4.30	58.74	64.89
C-CP-A_D_	10	19.22	1.25	18.33	20.11

**Table 2:** Analysis of variance (ANOVA) and contrast of difference of Vickers microhardness upon different anti-demineralizing materials at a different stage of application for enamel (2.1) and cementum (2.2) before material application (*B*_M_), after material application (*A*_M_), and after inducing demineralization (*A*_D_).

2.1 ANOVA of Vickers microhardness for enamel						
Source		SS	df	MS	F	*p*
Intercept		3119783.313	1	3119783.313	18306.787	0.001
Material		1163.047	2	581.524	3.412	0.048
Error		4601.252	27	170.417		

Contrasts of Vickers microhardness for enamel						
Source	Stage	SS	df	MS	F	*p*

Stage	B_M_ vs. A_M_	3931.014	1	3931.014	40.123	0.001
	A_M_ vs. A_D_	1541686.954	1	1541686.954	15185.474	0.001
Stage ^*∗*^ material	B_M_ vs. A_M_	2094.981	2	1047.490	10.692	0.001
	A_M_ vs. A_D_	2222.922	2	1111.461	10.948	0.001
Error	B_M_ vs. A_M_	2645.296	27	97.974		
	A_M_ vs. A_D_	2741.143	27	101.524		

2.2 ANOVA of Vickers microhardness for cementum						
Source		SS	df	MS	F	*p*

Intercept		67792.357	1	67792.357	4624.300	0.001
Material		230.227	2	115.113	7.852	0.002
Error		395.821	27	14.660		

Contrasts of Vickers microhardness for cementum						
Source	Stage	SS	df	MS	F	*p*

Stage	B_M_ vs. A_M_	348.298	1	348.298	9.835	0.004
	A_M_ vs. A_D_	60179.844	1	60179.844	3579.527	0.001
Stage ^*∗*^ material	B_M_ vs. A_M_	739.973	2	369.986	10.448	0.001
	A_M_ vs. A_D_	175.189	2	87.595	5.210	0.012
Error	B_M_ vs. A_M_	956.150	27	35.413		
	A_M_ vs. A_D_	453.930	27	16.812		

SS, sum of squares; MS, mean square; df, degree of freedom; F, F-ratio; *p*, *p* value.

**Table 3:** Multiple comparisons of Vickers microhardness of enamel and cementum upon application of nanohydroxyapatite (NHA) and Clinpro (CP) compared with no treatment (NT) at different stages including before material application (*B*_M_), after material application (*A*_M_), and after inducing demineralization (*A*_D_).

Stage	Enamel				Cementum			
A_M_–B_M_		NT	NHA	CP		NT	NHA	CP
	NT	1.000	0.002	0.001	NT	1.000	0.002	0.001
	NHA		1.000	1.000	NHA		1.000	1.000
	CP			1.000	CP			1.000

A_D_–A_M_		NT	NHA	CP		NT	NHA	CP
	NT	1.000	0.027	.001	NT	1.000	0.060	0.016
	NHA		1.000	.233	NHA		1.000	1.000
	CP			1.000	CP			1.000

## Data Availability

The data used to support the findings of this study are included within the article.
